# Annoyance and Worry in a Petrochemical Industrial Area—Prevalence, Time Trends and Risk Indicators

**DOI:** 10.3390/ijerph10041418

**Published:** 2013-04-03

**Authors:** Gösta Axelsson, Leo Stockfelt, Eva Andersson, Anita Gidlof-Gunnarsson, Gerd Sallsten, Lars Barregard

**Affiliations:** Department of Occupational and Environmental Medicine, University of Gothenburg, Box 414, Gothenburg S-405 30, Sweden; E-Mails: leo.stockfelt@amm.gu.se (L.S.); eva.m.andersson@amm.gu.se (E.A.); anita.gidlof@amm.gu.se (A.G-G.); gerd.sallsten@amm.gu.se (G.S.); lars.barregard@amm.gu.se (L.B.)

**Keywords:** petrochemical industry, industrial noise, industrial air pollution, annoyance, worry

## Abstract

In 1992, 1998, and 2006, questionnaires were sent to stratified samples of residents aged 18–75 years living near petrochemical industries (n = 600–800 people on each occasion) and in a control area (n = 200–1,000). The aims were to estimate the long-term prevalence and change over time of annoyance caused by industrial odour, industrial noise, and worries about possible health effects, and to identify risk indicators. In 2006, 20% were annoyed by industrial odour, 27% by industrial noise (1–4% in the control area), and 40–50% were worried about health effects or industrial accidents (10–20% in the control area). Multiple logistic regression analyses revealed significantly lower prevalence of odour annoyance in 1998 and 2006 than in 1992, while industrial noise annoyance increased significantly over time. The prevalence of worry remained constant. Risk of odour annoyance increased with female sex, worry of health effects, annoyance by motor vehicle exhausts and industrial noise. Industrial noise annoyance was associated with traffic noise annoyance and worry of health effects of traffic. Health-risk worry due to industrial air pollution was associated with female sex, having children, annoyance due to dust/soot in the air, and worry of traffic air pollution.

## 1. Introduction

Emissions from industrial plants may give rise to both annoyance and concerns about serious health effects. In the petrochemical industry, potentially harmful substances are produced or used and some are toxic, foul smelling, or flammable. Several reports show increased risk of cancer and low birth weights in populations living close to petroleum-polluted areas [1,2,3,4], while others show no such significant relationships [5,6,7,8]. Self-reports of various symptoms and of asthma have been related to proximity to petrochemical industries [9,10,11,12]. Stable petrochemical production may be associated with low, but long-term, exposure to industrial emissions. However, accidents in the plants or during transportation and disruption of production may temporarily cause high levels of exposure in the population living near to those plants. 

In Sweden, where this study was conducted, the petrochemical industry is located on the western coastline, with particular concentration around the municipality of Stenungsund. The local authorities, the public, and the industry in Stenungsund have requested risk evaluations and health surveillance in the municipality, and studies of cancer and miscarriage rates have been conducted over the past few decades [5,8]. No relationship, however, has been found between residence near petrochemical plants and increased risk of cancer or miscarriage.

Although concentrations of emissions from petrochemical plants are generally low, these substances often pollute the surrounding neighbourhoods with odours. Odour is an environmental stressor affecting people’s quality of life [12,13,14], and annoyance due to odour *per se* and its association with perceived health status (self-reported symptoms) have been thoroughly studied among people living close to various industrial odour sources e.g., [13,14,15,16,17,18,19]. Fewer studies of this type have been conducted in residential areas located close to oil refineries or petrochemical industries [9,20,21,22,23]. The studies showed moderate to strong associations between odour exposure (frequency, odour concentration), odour perception (perceptibility, pleasantness/unpleasantness), and degree of odour annoyance. The two latter variables have been found to mediate the relationship between odour exposure and symptom reporting. The concept of annoyance is further considered in relation to environmental stress below.

Besides emitting airborne substances, petrochemical industrial sites and their associated means of transport (heavy vehicles, tank trucks, railways, ships) can cause chronic exposure to noise to those working or living in close proximity. Peak noise levels produced periodically from the burning of surplus gases may be perceived by the inhabitants of the surrounding neighbourhoods. Field studies of annoyance with community noise from stationary sources are rare [24]. Miedema and Vos [25] presented dose-response curves relating noise levels (DENL: day-evening-night levels) to annoyance based on data from a large study (N = 1,875) at 11 stationary sources (industries producing chemicals, metal, paper, ammonium, etc., and shunting yards). Noise from shunting yards caused more annoyance than other industrial noises at the same sound level, however, a review of the adverse effects of industrial noise concludes that factors other than the sound pressure level are important to people’s reactions [24]. Impulsive noise and tonal components (e.g., high tones) cause considerably higher annoyance than noise from other sources, as demonstrated by the findings of a field study investigating noise effects in areas close to petrochemical industries [26]. In addition to the noise exposure *per se*, many non-acoustic factors influence the extent of noise annoyance [27,28].

Odour and noise exposure are both ambient environmental stressors that may have adverse effects on health, well-being, and quality of life [12]. Many of these effects are similar and are influenced, overall, by the same modifiers. The most frequently reported effect of these exposures is annoyance. Annoyance can be defined as a feeling of displeasure associated with any agent or condition believed to have an adverse effect [29]. Annoyance is coupled with feelings of irritation, frustration, dissatisfaction, discomfort, distress, anger, fear, and hatred [30]. Common modifiers for odour and noise annoyances are related to constitutional and contextual factors (age, children in the family, sensitivity, health status), attitudes towards and dependency upon the source of exposure, worry or fear about health effects, perceived control, coping capacity, other environmental stressors, mistrust of source authorities, expectations of an increasing level of exposure, concerns about loss of property value, and warnings in the media about environmental pollution [23,30,31,32,33]. 

The primary aim of this study was to estimate the long-term occurrence and potential change over time of annoyance due to industrial odour and industrial noise and of worry about health effects in a population living close to petrochemical industries. The secondary aims were to find out whether specific groups in the population were more annoyed or worried than others. 

## 2. Methods

### 2.1. Study Area

The petrochemical industry around the municipality of Stenungsund is the largest in Sweden. It was established between 1963 and 1980 and had about 2,000 employees in 2005. The industrial complex, which operates 24 h a day, includes a large naphtha cracker to produce ethylene and propene, and other plants that produce polyethylene, polyvinyl chloride (PVC), amines, surfactants, and oxo-alcohols. The ambient air is polluted by a large spectrum of compounds, including established or suspected carcinogens such as ethylene, benzene, 1,3-butadiene, propene, ethylene oxide, and vinyl chloride (for more detailed information about these exposures, see [8]. Some of these compounds cause odours *per se*, but odours can also be produced by the industrial processes. 

The petrochemical industry in this area has five large torches or chimneys. Burning surplus gases can occasionally cause sooty flares and a rumbling noise. Because the industrial sites in Stenungsund handle large quantities of flammable substances, safety systems sometimes cause internal alarms to go off and the alarm system is tested each month. Transportation to and from the industrial plants use heavy vehicles, tank trucks, railway cars, and ships. Some of the road transport carries hazardous materials. The railway that carries various hazardous materials goes right through the community. To minimize railway accidents, all shunting is done in a special shunting yard. 

**Figure 1:**
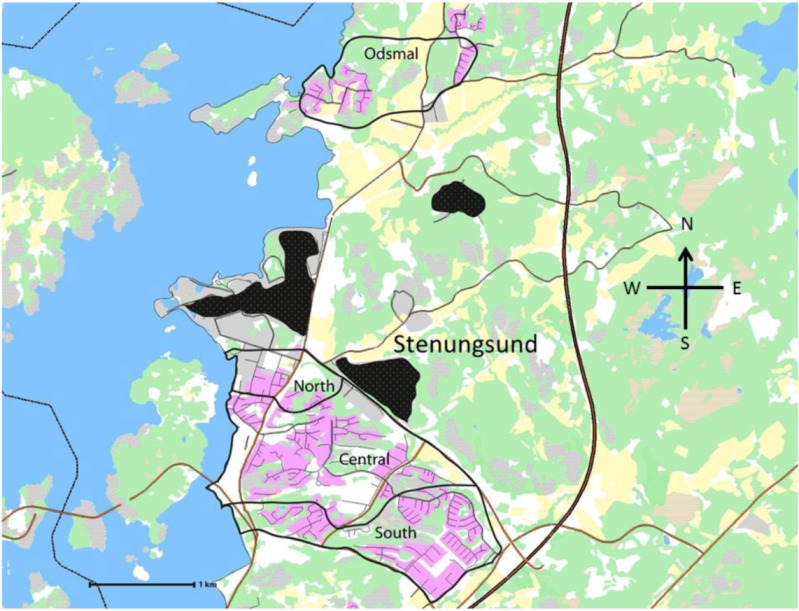
“Exposed areas”: three areas (North, Central, and South) in the municipality of Stenungsund and the municipality of Odsmal, 3 km north of Stenungsund in the direction of the prevailing winds from the industrial sites. The black areas represent the location of the petrochemical plants. Residential areas are marked with pink colour.

Four residential areas located relatively close to the petrochemical industrial sites were included in the study (Figure 1). These areas are here called exposed areas and consist of the north, central, and south areas of the municipality of Stenungsund and the municipality of Odsmal, 3 km north of Stenungsund and exposed to the prevailing south and south-west winds from the Stenungsund industries. The environment within and around these municipalities is known for its natural beauty (archipelago, lakes, and forests) and great opportunities for outdoor activities (e.g., sailing, hiking, bathing). Despite the petrochemical industry’s distinct presence, the area is a very popular region for settlement.

### 2.2. Study Population

The target population was defined as every person aged 18 to 75 years who had been living for at least one year in one of the four specified exposed areas at different distances from the petrochemical industrial sites (Figure 1). The control population was recruited from Kungalv, a town about the same size as Stenungsund, but without petrochemical industries, located 24 km south-east of Stenungsund and therefore not considered exposed to emissions from the petrochemical plants.

Sampling from the target population in Stenungsund (stratified sampling, four sub-areas) and from the control population was conducted on three separate occasions: 1992, 1998, and 2006. In 1992 the sample was taken from the municipality census register of 1990 and included 764 persons from Stenungsund and Odsmal and 854 from Kungalv. On the second occasion (1998), the sample was taken from the National Central Bureau of Statistics national population register. In all, 855 people from Stenungsund and Odsmal and 976 from Kungalv were selected. The sample in 2006 was also taken from the National Central Bureau of Statistics and included 554 persons from Stenungsund and Odsmal and 198 from Kungalv. If there were two or more people aged 18 to 75 years in a household, only one person was selected (the one whose birthday was closest to the study period).

### 2.3. Questionnaire

Postal questionnaires were sent to the selected persons with a letter explaining the background and the purpose of the study and assuring potential participants that participation was anonymous and voluntary. According to the ethics committee of the University of Gothenburg, this type of questionnaire did not require ethics committee approval. Two reminders were sent to those who did not respond.

The questionnaire contained four sections: (i) personal information (age, sex, marital status, children living at home, and employment); (ii) housing conditions (housing type, years of residency, housing satisfaction); (iii) annoyance with various environmental sources of exposure; (iv) worries about health effects or accidents from various environmental sources of exposure. 

For questions in sections (iii) and (iv), we asked the participants to specify how much they were annoyed and worried by seven sources of exposure (including but not emphasizing exposure from the petrochemical industry). The questions about annoyance asked about road traffic noise, exhaust from motor vehicles, industrial noise, industrial odour, dust or soot in the air, pollution from residential wood burning, and noise from neighbours, and were phrased as “Are you annoyed/disturbed by any of the following, when you are in or around your home?” The answers were scored on a 3-point category scale, “Not annoyed/disturbed”, “Annoyed/disturbed”, and “Very annoyed/disturbed”. The five questions about worry asked about risk of accident due to road traffic, or railway traffic, risk of health effects due to air pollution from traffic or from industry, and risk of accidents due to industrial activity. The questions were phrased as “How often do you experience worry, concerning yourself or your family, about…”. The answer was scored on a 3-point category scale: “Never or almost never”, “Sometimes or periodically”, and “Daily or almost daily”. 

### 2.4. Background Data

Table 1 shows the response rate, sex, age, type of housing, number of years living in the present dwelling, marital status, employment status (for 2006 also including work in petrochemical industry), having children living at home and housing satisfaction on the three survey occasions. The response rate in the exposed area was consistent at 72–74% and in the control area at 69–72%. The participants were on average 6 years older in the last survey than those in the first survey, in both the exposed and the control areas. The differences between the two areas in proportions of married people, women, children living at home, and students were generally quite small. The proportion of working people was somewhat higher and the fraction of retired people lower in the exposed area than in the control area. One major difference between regions was in housing: the proportion of people living in single family houses was more than twice as high in the exposed area than in the control area. Number of years living at the present address (mean values) did not differ between the exposed and the control areas. 

**Table 1:** Background data on the respondents in the exposed area and the control area, on three occasions.

	Exposed area (4 sub-areas)			Control area (Kungalv)		
	1992	1998	2006	1992	1998	2006
Sample size	764	855	554	854	976	198
- North	71	100	108			
- Central	216	295	148			
- South	253	292	149			
- Odsmal	224	168	149			
Response rate (%)	74	72	74	71	69	72
Women (%)	51	53	56	45	52	62
Age (mean)	45	48	51	46	47	52
Type of housing (%)						
- Rent apartment	17	14	13	27	29	26
- Co-operative apartment	6	11	17	22	21	26
- Row house	13	13	9	23	21	21
- Detached house	64	62	61	29	28	27
Years in present house/apartment (mean)	11	13	14	12	12	14
Married/living together (%)	71	69	70	70	65	65
Employment (%)						
- Gainfully employed	71	68	64 *	63	67	59
- Students/housewives	10	6	4	10	7	4
- Retired	16	22	28	23	22	35
- Unemployed	2	4	4	3	4	1
Children living at home (%)	42	34	31	37	35	28
Housing satisfaction (%)						
- Very good/good	92	97	95	95	95	98

* 16% were employed in the petrochemical industries.

### 2.5. Statistical Methods

Annoyance due to industrial odour and industrial noise and worry about the possible health effects of industrial air pollution or industrial accidents was calculated for each year as crude prevalence rates and crude odds ratios. The development of annoyance and worry over time (1992, 1998, and 2006) was investigated, as well as which factors were associated with each annoyance and worry variable, respectively. In multiple logistic regression analyses, the log-odds of being annoyed (or being worried) were modelled as a linear function of the explanatory variables. From the logistic regression, the prevalence can be estimated as:

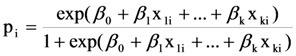
(1)
where p = probability of being annoyed (or worried), index i refers to person i, and index k refers to the number of explanatory variables (X_1_, X_2_, ....X_k_) included in the logistic regression. We wanted an adjusted comparison of the prevalence for the three years, and two different regression models were estimated. Model A included the background variables year, sex, age, type of residence, number of years living in present residence, children living at home, and employment status. Model B was selected using backward elimination and included year, gender and age, together with those among the other background variables and variables for worry (or annoyance) which showed a significant association with the outcome. These variables were considered as potential confounders in the association between time and prevalence. When any two of the potential explanatory variables were highly correlated, only one of the variables were included in the multiple regression model, in order to avoid problems with multicollinearity. The results of the regressions are presented as adjusted odds ratios (OR) and mean adjusted prevalence rates. When using a logistic regression, the effect of e.g., X_1_ on the prevalence depends on the values of the other X-variables. An adjusted mean prevalence for each year is calculated on the assumption that the distribution of the X-variables (except year) would be the same in 1998 and 2006 as it was in 1992 (*i.e.*, using data from 1992 as reference distribution).

Since the exposed area consisted of four sub-areas and the samples sizes were not proportional to the population, weighted analyses were performed and the proportion of those annoyed (worried) in the entire exposed area was estimated as:

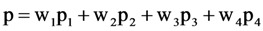
(2)
where e.g., w_1_ is the proportion of the whole exposed population living in sub-area 1 and p_1_ is the proportion of annoyed (worried) in area 1. Also, in the logistic regression, weighting was used. The analyses were made using SAS 9.1.3 and p-values below 0.05 were considered significant. 

## 3. Results

**Figure 2:**
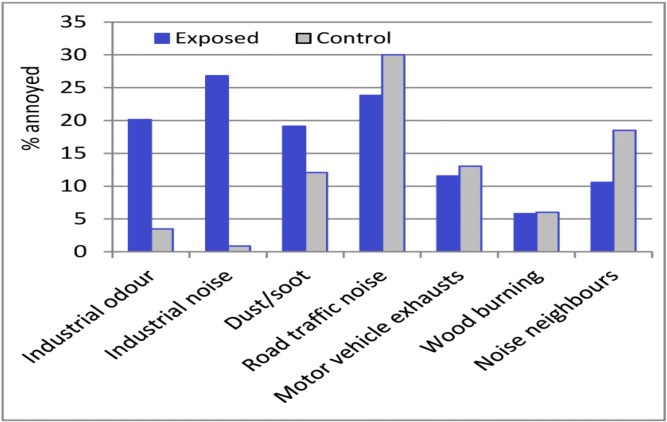
Prevalence of annoyances in 2006, in the exposed area (results from North, Central, and South Stenungsund, and Odsmal weighted together) and in the control area (Kungalv).

The prevalence of annoyance in 2006 for different sources of exposure in the entire exposed area close to the petrochemical industries and the control area is shown in Figure 2. The largest difference was seen for industrial noise, with 27% (95% CI 20–34%) of the respondents in the exposed area reporting that they were annoyed or very annoyed compared with 1% (95% CI 0–3%) in the control area. There was also a striking difference between the exposed and control areas in annoyance due to industrial odour: 20% (95% CI 14–26%) *vs*. 3.5% (95% CI 0.2–6.8%). The differences between the areas were smaller for annoyance due to dust or soot. For other sources (wood burning, road traffic noise, motor vehicle exhaust, and noise from neighbours) the proportions of annoyance were somewhat higher in the control area than in the exposed areas. The higher prevalence of annoyance due to noise from neighbours in the control area is due to a higher proportion living in rent and co-operative apartments. One probable reason for the high prevalence of annoyance from road traffic noise is that a highway runs through the town. 

### 3.1. Annoyance due to Industrial Odour

**Figure 3:**
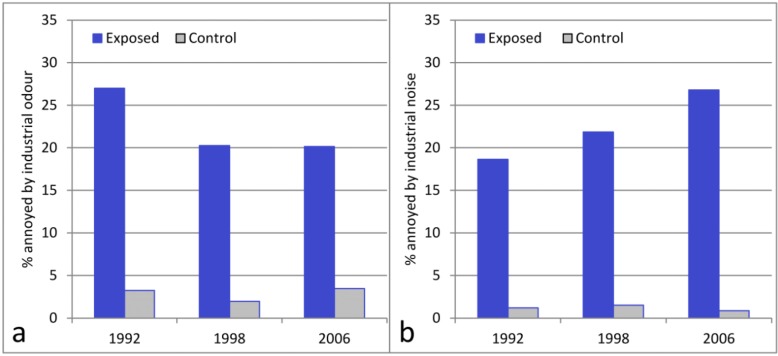
Prevalence of annoyance. The results from the exposed areas (North, Central, and South Stenungsund, and Odsmal) are weighted together and compared to the control area (Kungalv). Annoyance from industrial odour (**a**); annoyance from industrial noise (**b**).

Annoyance due to industrial odour (the proportion of annoyed or very annoyed) in the exposed area was highest in 1992 (27%, 95% CI 22–32%) and lower in 1998 (20%, 95% CI 16–24%) and 2006 (20%, 95% CI 14–26%). This decrease in annoyance by odour from 1992 to 1998 was significant for the crude OR as well as for the adjusted OR for both Model A (including background variables only, see Section 2.5) and model B in the logistic regression analyses (Table 2). In the control area the proportion of annoyance was low (2–4%) in all surveys (Figure 3). 

**Table 2:** Annoyance and worries about aspects of industrial activity over time in the exposed area.

		Crude		Adjusted		
	Year	OR (95% CI)	Prevalence	OR * (95% CI)	OR ^†^ (95% CI)	Mean prevalence ^†^
Annoyance	1992	1.00	27%	1.00	1.00	24%
industrial odour	1998	0.69 (0.51–0.92)	20%	0.68 (0.50–0.91)	0.69 (0.50–0.96)	19%
	2006	0.68 (0.51–0.91)	20%	0.72 (0.53–0.99)	0.61 (0.42–0.88)	17%
Annoyance	1992	1.00	19%	1.00	1.00	18%
industrial noise	1998	1.22 (0.90–1.66)	22%	1.22 (0.89–1.65)	1.36 (0.97–1.90)	23%
	2006	1.60 (1.19–2.15)	27%	1.85 (1.36–2.53)	1.53 (1.08–2.16)	25%
Worry	1992	1.00	51%	1.00	1.00	49%
health effects ^‡^	1998	0.93 (0.73–1.19)	49%	0.96 (0.74–1.23)	1.06 (0.78–1.42)	50%
	2006	1.04 (0.82–1.34)	52%	1.12 (0.86–1.47)	0.98 (0.70–1.37)	48%
Worry	1992	1.00	42%	1.00	1.00	43%
accidents ^§^	1998	0.83 (0.65–1.07)	38%	0.84 (0.65–1.09)	0.76 (0.55–1.06)	39%
	2006	0.98 (0.76–1.26)	42%	0.96 (0.74–1.27)	0.86 (0.62–1.22)	41%

* Estimated from logistic regression model including all background variables (Model A, see statistical methods Section 2.5); ^†^ Estimated from the logistic regression Model B, see Table 3; ^‡^ Worry about health effects of industrial air pollution; ^§^ Worry about accidents due to industrial activity.

**Figure 4:**
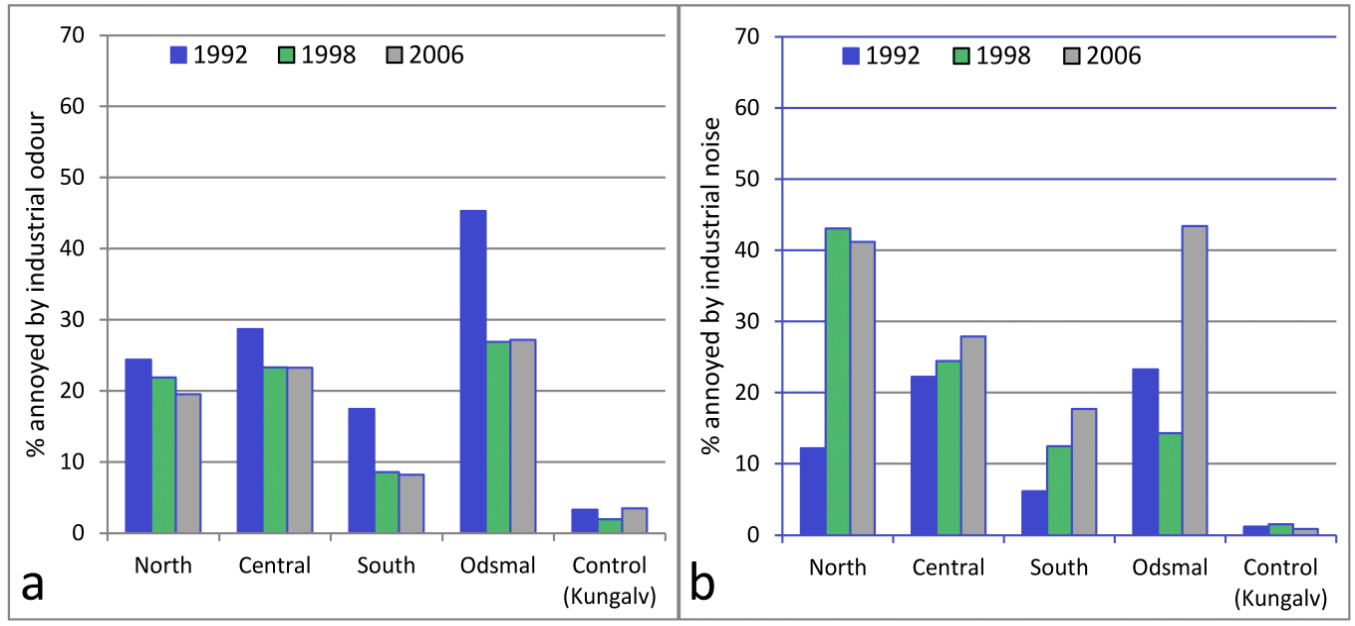
Prevalence of annoyance for each exposed area (North, Central, and South Stenungsund, and Odsmal) and the control area (Kungalv). Annoyance from industrial odour (**a**); annoyance from industrial noise (**b**).

Figure 4 shows that a decrease of odour annoyance occurred between 1992 and 1998 in all four sub-areas within the exposed area. The highest proportion of annoyance due to odour was found in Odsmal in all three surveys and the lowest was in the southern area of Stenungsund. There was no change in these two sub-areas between 1998 and 2006. 

Table 3 shows the results (adjusted odds ratios with 95% CI) of the logistic regression Model B for industrial odour annoyance as the outcome variable. Of the background variables, only sex has a significant association with odour annoyance (women were more likely to be annoyed than men). Annoyance due to industrial noise had the strongest association with odour annoyance, OR = 7.6 (5.5–10.5), followed by worry of health effects due to industrial air pollution and annoyance due to motor vehicle exhaust. 

**Table 3:** Odds ratios (OR) for the variables associated with each outcome in the exposed area (Model B).

		Annoyance industrial odour	Annoyance industrial noise	Worry health effects industrial air pollution	Worry accidents industrial activity
		OR (95% CI)	OR (95% CI)	OR (95% CI)	OR (95% CI)
Year	1998 (ref = 1992)	0.62 (0.44–0.89)	1.65 (1.14–2.37)	n.s.	n.s.
	2006 (ref = 1992)	0.51 (0.35–0.75)	2.48 (1.71–3.59)	n.s.	n.s.
Sex	Female	1.44 (1.05–1.96)	0.63 (0.47–0.86)	1.92 (1.48–2.49)	1.63 (1.23–2.16)
	(ref = male)				
Age (continous)	10-year effect	n.s.	n.s.	n.s.	n.s.
	(old *vs*. young)				
Type of housing	Rent apartment	n.s.	0.63 (0.44–0.90)	n.s.	n.s.
	Co-op apartment (ref = row house/ detached house)	n.s.	0.33 (0.20–0.55)	n.s.	n.s.
Years in present house (continous)	5-year effect (longer *vs*. shorter time)	n.s.	n.s.	0.91 (0.84–0.98)	0.89 (0.82–0.97)
Employment	Gainfully employed	n.s.	n.s.	n.s.	2.48 (1.80–3.43)
	(ref = student, retired, working at home, unemployed)				
Have children living at home	Yes	n.s.	0.66 (0.47–0.93)	1.46 (1.09–1.95)	n.s.
	(ref = no)				
Housing satisfaction	Not good/bad	n.s.	n.s.	2.10 (1.17–3.76)	n.s.
	(ref = very good/ good)				
Annoyed by motor vehicles exhaust	Very annoyed/ annoyed	2.53 (1.65–3.86)	not included	n.s.	not included
	(ref = not annoyed)				
Annoyed by traffic noise	Very annoyed/ annoyed	not included	2.77 (1.99–3.86)	not included	not included
	(ref = not annoyed)				
Annoyed by industrial odour	Very annoyed/ annoyed	not included	7.95 (5.73–11.03)	3.91 (2.74–5.59)	n.s.
	(ref = not annoyed)				
Annoyed by industrial noise	Very annoyed/ annoyed (ref = not annoyed)	7.61 (5.54–10.45)	not included	not included	not included
Annoyed by dust/soot in the air	Very annoyed	not included	not included	9.36 (1.96–44.8)	not included
	Annoyed	not included	not included	2.46 (1.63–3.70)	not included
	(ref = not annoyed)				
Worried health effects traffic air pollution	Worried daily/ sometimes	n.s.	not included	12.9 (8.88–18.9)	not included
	(ref = almost never)				
Worried health effects industrial air pollution	Worried daily	6.40 (3.68–11.12)	3.23 (1.87–5.59)	not included	76.7 (30.1–163)
	Worried sometimes	3.79 (2.68–5.35)	1.81 (1.30–2.51)	not included	14.3 (10.6–19.2)
	(ref = not annoyed)				
Worried accidents train traffic	Worried daily/ sometimes	not included	not included	not included	1.91 (1.09–3.35)
	(ref = almost never)				

### 3.2. Annoyance due to Industrial Noise

Figure 3 shows that annoyance due to industrial noise (the proportion of annoyed or very annoyed) has increased in the exposed area from 19% (95% CI 14–23%) in 1992 to 27% (95% CI 20–34%) in 2006. In 1998, this proportion was 22% (95% CI 18–26%). This increase in annoyance with industrial noise from 1992 to 2006 was significant for both the crude OR and the adjusted ORs with both models A and B in the logistic regression analysis (Table 2). In the control area the proportion of annoyance remained at a constant low level (1–2%).

Figure 4 shows that the proportion of annoyance with noise was higher in the north area located closest to the industries in 1998 and 2006 than in the central and southern areas. In 2006, the prevalence of annoyance was highest in the areas of Odsmal (43%, 95% CI 37–50%) and north Stenungsund (41%, 95% CI 36–46%). In Odsmal, the prevalence rate was three times higher in 2006 than in 1998, while in northern Stenungsund it was similar to the prevalence in 1998. In the area of south Stenungsund, 1 to 4 km from the industries, the rate of annoyance was 18% (95% CI 11–24%) in 2006.

Table 3 lists the adjusted odds ratios of the logistic regression Model B for annoyance with industrial noise as the outcome variable. Two of the background variables were significantly associated with annoyance with industrial noise. These were sex (men were more likely to be annoyed than women) and type of house (inhabitants living in a row house or a detached house were more likely to be annoyed with noise than those living in a co-operative apartment). In addition to the strong association with annoyance of industrial odour, associations were also seen with worry about the possible health effects of air pollution from industry, OR = 3.2 (1.9–5.6) and annoyance with traffic noise, OR = 2.8 (2.0–3.9).

### 3.3. Worry about Health Effects due to Industrial Air Pollution

**Figure 5:**
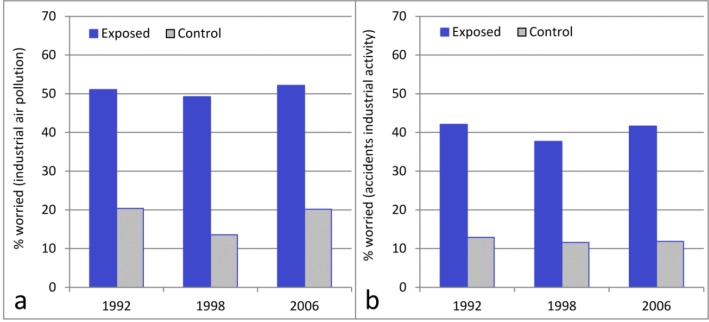
Prevalence of worry about industrial activity. The results from the exposed areas (North, Central, and South Stenungsund, and Odsmal) are weighted together and compared with the control area (Kungalv). Worry about health effects of industrial air pollution (**a**); worry about accidents from industrial activity (**b**).

**Figure 6:**
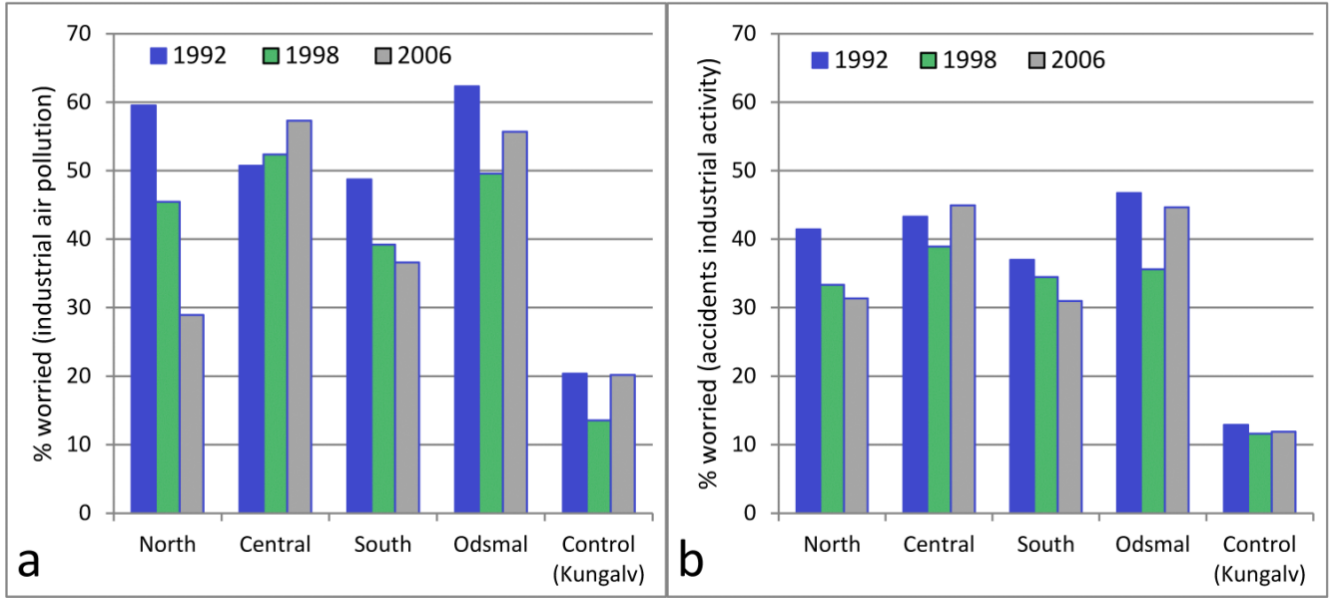
Prevalence of worry about industrial activity for each exposed area (North, Central, and South Stenungsund, and Odsmal) and the control area (Kungalv). Worry about health effects of industrial air pollution (**a**); worry about accidents caused by industrial activity (**b**)*.*

Approximately 50% of the respondents in the exposed areas reported that they sometimes/periodically or daily/almost daily worried that industrial air pollution may affect their own or their relatives’ health (Figure 5). Seven percent reported daily or almost daily worry. There was no significant change in worry about industrial air pollution over time in either models A or B (Table 2). In the control area the prevalence rate varied between 13% (95% CI 11%–16%) and 20% (95% CI 17–24% in 1992 and 13%–27% in 2006) on the three surveys occasions. Worry about health effects due to industrial air pollution was not clearly related to distance of residence from the industrial site (Figure 6). The proportion reporting concern in the four exposed sub-areas varied from 49% in area south to 62% in Odsmal in 1992. In 2006, this proportion varied from 29% in area north to 57% in the central area.

Table 3 shows the adjusted odds ratios of the logistic regression model B for worry about health effects due to industrial air pollution as the outcome variable. Significant background variables were sex (women were likely to be worried more often than men), having children living at home, dissatisfaction with the residential area and living short time in the present house. Worrying about the possible health effects of air pollution from traffic was very strongly associated with worry about possible health effects of industrial air pollution, OR = 12.9 (8.9–18.9). Significant associations were also found for annoyance due to dust/soot in the air and industrial odour. 

Data from the survey in 2006 showed that the proportion of people who worried was significantly lower among employees of the petrochemical industries (35%) than among people employed elsewhere (60%). Because we had no such data in the surveys conducted in 1992 and 1998, this variable was not included in the logistic regression model.

### 3.4. Worry about Accidents due to Industrial Activity

In the exposed areas, approximately 40% of the respondents reported that they sometimes/periodically or daily/almost daily worried about accidents due to industrial activity (Figure 5). Four percent reported daily or almost daily worry. According to the logistic regression Models A and B (Table 2), there was no significant change in worry about industrial accidents over time. 

In 2006, the prevalence rate was somewhat higher in the central area of Stenungsund and in Odsmal (45%) than in the northern and southern areas of Stenungsund (32%). In 1998, the differences between the four areas were very small (Figure 6). 

Table 3 shows the adjusted odds ratios of model B for the outcome variable of worry about accidents due to industrial activity. Three of the background variables were significantly associated with worry about industrial accidents. These were sex (women were more often worried than men), living in the area for a shorter time, and being gainfully employed, OR = 2.5 (1.8–3.4). The strongest association in the model was with worry about the possible health effects of industrial air pollution, OR = 77 (30–163). Among those not worried about the possible health effects of industrial air pollution, the prevalence of worry about industrial accidents was 12%, whereas it was 91% among those who were worried about the health effects of industrial air pollution. Another significant variable in the model was worry about accidents due to train traffic. Data from the 2006 survey among employed people showed that the proportion who were worried about industrial accidents was significantly lower among people employed in the petrochemical industries (33%) than in people employed elsewhere (47%). 

## 4. Discussion

The present longitudinal study reveals how inhabitants react to the situation of living in close vicinity to petrochemical industries. The strength of the study design is that the results are based on three surveys that were conducted in 1992, 1998, and 2006 using the same questionnaire. This type of study is rare c.f. [22], but it has several benefits. Since the same variables are measured repeatedly across time, changes in self-reported health and well-being in the population, which may be related to measures undertaken to reduce exposure (e.g., odour) or other changes in the petrochemical industry (e.g., increase of production), may be evaluated. Although 1992 was used as a reference year in the present study, we know from unpublished data from the same study sites that annoyance with industrial odour was more prevalent in the 1980s than in recent years. Annoyance with industrial noise has varied more within each site and decade.

Changes over time in the population, such as the proportion of males to females, mean age, number of years living in present residence, and employment status were taken into account in the statistical analyses. From 1992 to 2006 the population increased by approximately 10%, and it is possible that this increase varied within the exposed area. 

### 4.1. Annoyance with Industrial Odours

Studies of industrial odour may be conducted as epidemiological surveys or panel surveys. Persson *et al*. [21] reported in a panel study conducted close to a petrochemical site in Finland that complaints about odour occurred most frequently at low wind velocities. Most of the observations by the panel were made from January to April and early in the morning. The episodes of offensive odour amounted to only 1% of the total observation time. However, there may be discrepancies between the experience of odours in a group of panellists and the experience of the general population. Sucker *et al*. [18] found that odours from six different industrial sources were reported as more intense and more unpleasant by residents than by panellists. 

In our study we have data on the prevalence and the degree of annoyance caused by odour, but no information about the type or level of odorous airborne emissions at the time of the surveys. The total emissions of VOC from the petrochemical plants have been estimated by the municipal environmental authority to approximately 2,100 tons in 1993, 1,770 tons in 1998 and 1,600 tons in 2006. It is likely that exposure to odorous compounds and noise from the industrial plants is lowest in the most distant sub-area (south) and highest in the areas located closest to the plants or in the prevailing wind direction from the plants. However, it was not possible to classify all the sub-areas as proxies for exposure, since two of these (north, central) are in part located close to the plants and one (Odsmal) is located in the prevailing wind direction. Furthermore, we had no data on how often and what time of the day people experienced odours or were annoyed or how they perceived the odours (e.g., pungent, irritating). This would have been helpful because the odour episodes have temporal variations and the literature shows a relationship between the perception of unpleasant odours and annoyance with odours e.g., [9,13,16,23] The crude prevalence of annoyance with odour from industry was 20% in the exposed area in 1998 and 2006 *versus* 27% in 1992. This proportion of annoyed inhabitants in the exposed areas were considerably higher than the few percent reported in the control area on the three survey occasions. The annoyance was most frequent in the sub-area in the prevailing wind direction from the industries (Odsmal) and least frequent in the most distant sub-area (south), which is in line with previous findings [9,11,22]. The decrease between 1992 and 1998, which was observed in all sub-areas but was largest in areas south and Odsmal, was statistically significant and probably a consequence of the emission reduction measures that were undertaken in the mid-1990s [8]. Luginaah *et al*. [22] reported from a similar study in Canada that active steps to reduce emissions from the petroleum industry lowered the prevalence of annoyance with odour. 

Previous research shows that problems with odours seem to be more common among certain groups. We found that women were more likely than men to be annoyed by industrial odour and sex differences in sensory and perceptual abilities have been found for olfaction with females being more sensitive than males [13,34]. Those who have respiratory or cardiovascular diseases, or are sensitive to odours or exhibit a generalised tendency to report annoyance or sensitivity across divergent environmental conditions (e.g., temperature, dust, various noise sources), and people with neuroticism/negative affectivity [13,35] are also more affected by odours. In the present study, those who were annoyed by vehicle exhaust or industrial noise or who believed that air pollution from industry affected their health were also more likely to be annoyed by industrial odour. This result may indicate a group who is more exposed than others to environmental exposures and/or potentially a group vulnerable for various environmental stressors. We found no indication that those with potentially limited socio-economic resources (student/retired/unemployed, renting an apartment) or those with children at home were more annoyed by industrial odours. Numbers of years living in the present dwelling was not significant, which may indicate that the inhabitants in the community do not become accustomed to the odours over time. This is similar to results reported by Luginaah *et al*. [23].

### 4.2. Annoyance with Industrial Noise

No estimation of noise levels from the industries was done at the time of the three survey occasions. However, two investigations of the industrial noise were conducted in 1986 and 2008 in the north and central areas. Compared with the 1986 investigation, the equivalent noise levels (dBA) have generally decreased slightly in 2008 [36]. In the area north and the half part of area central that is located closest to the petrochemical industry, the estimated industrial noise exposure during the night varies between about L_night22-07_ 40–55 dB (for a worst case scenario based on the assumption that there is a downwind from all noise sources simultaneously [36]. Night noise levels are the most important to estimate because traffic noise in most settings dominates during the day and masks the noise from the industries (about 9–10 dB difference in traffic noise levels between day and night). When night noise levels are above 42 dB outside the bedroom window, the risk for adverse health effects (e.g., sleep disturbances and insomnia) increases [27]. But daytime noise from the industries may also be less masked and heard more clearly during the weekends when the traffic intensity and the noise are lower. This may disturb various daily activities particularly in the summer when people more often have their windows open and are spending more time outdoors. Pierette *et al*. [37] found that the annoyance due to industrial noise was not consistent but changed depending both on the time of the day and type of season. However, since it was not possible to link noise levels to each participant’s home in the present study, it should be noted that the calculated levels from the petrochemical industries in 2008 only give a crude and overall picture of the noise situation in the area north and central.

Despite the slight decrease of noise levels between 1986 and 2008, there was an increase over time in the prevalence of annoyance with industrial noise, in contrast to the decrease over time in annoyance with odours. This increase was statistically significant between 1992 and 2006. In 2006, approximately 25% of respondents in the exposed areas reported annoyance with industrial noise compared with only two percent in the control area. There was a significant increase in annoyance with noise in the sub-areas north Stenungsund from 1992 to 1998 and Odsmal from 1998 to 2006. A follow-up in Odsmal, two years later indicated no change in the prevalence of noise annoyance since 2006. One possible explanation of the very high prevalence of annoyance with noise in Odsmal, located about 3 km from the largest petrochemical industrial complex but in the main direction of the prevailing winds, is the replacement of the torch tops in two of the nearby industrial plants in 2000. This led to more frequent episodes of burning off surplus gases and peak noise levels occurring any time of the day and night. Previous studies have shown that impulsive noise with a sudden onset and termination, such as the release of gas or steam, increases annoyance [24,25,26,27]. A strong correlation between the direction of the prevailing winds from a chemical plant and the number of noise complaints has also been shown [26]. The high proportion of people annoyed by noise in the northern sub-area may also be partly explained by the fact that the largest of the five industrial torches could be seen and heard from that sub-area. It is also possible that certain industrial activity (e.g., industrial machinery, compressors) has increased the exposure of low frequency noise, which causes greater annoyance reactions than other noises at comparable sound pressure levels [27].

In most studies on community noise (traffic and industrial noise), sex had no influence on annoyance [38]. However, in the present study, men were more likely to be annoyed by industrial noise than women. The logistic regression model revealed that people living in a row house or a detached house were more likely to report annoyance with industrial noise than those living in a co-operative apartment. Because no detailed information on noise levels for the participants’ residences was available, we were unable to determine whether the difference in annoyance between people living in either of these types of housing was due to differences in industrial noise exposure. 

Annoyance with industrial noise was associated with traffic noise annoyance. This association is reasonable as parts of the study areas are located close to major roads and almost one in four of the respondents were annoyed by road traffic noise. However, noise sensitivity—a personality trait with heightened vulnerability to noise in general and a strong predictor of noise annoyance [28,30,39], may also have affected the finding. Although we did not assess noise sensitivity in our study we know from other questionnaire studies that a fairly large amount of the population (about 1/3) consider themselves to be quite sensitive or very sensitive to sounds or noise [40,41]. There was also an association between annoyance of industrial noise and industrial odour and worry about air pollution from industry. This latter finding is supported by results from a recent study that show a high positive correlation between fear of the industrial site and noise annoyance as well as with noise sensitivity [37].

### 4.3. Worry about the Possible Health Effects of Industrial Air Pollution

People living near petrochemical industries are often concerned about the possible health effects of air pollution [22,23,42,43]. Despite the fact that no relationship has been demonstrated between living near petrochemical industries in Stenungsund and health effects such as cancer [8] or miscarriage [5], we found that approximately half of the inhabitants in the exposed areas were sometimes/periodically or more often worried that industrial air pollution may have effects on their own or their relatives’ health. This was more than twice as common as in the control area. This may be because the public does not know about these studies or that there are other health effects that people are worried about. 

Luginaah *et al* [23] has presented an analytical model of health impacts of a refinery based on theoretical frameworks by others [14,16,44]. In this model belief that a refinery impacts health is considered as an environmental stressor mediating the association between odour annoyance and ill-health.

We are not aware of any other longitudinal studies close to petrochemical industries where the prevalence of worry about the possible health effects of industrial air pollution has been investigated over as long time as fifteen years. In addition to the high prevalence rate, it is worth noting that the overall proportion of worried inhabitants has not changed over the years in spite of reduced emissions and a lower prevalence of odour annoyance.

Similar results have been reported by Luginaah and colleagues [43] from in-depth interviews conducted in a petrochemical industrial area in Canada. Although residents reported a reduction in odours from the refinery, some people continued to be worried about health effects of invisible and odourless emissions. Children’s health (both short- and long-term) was one of the most important health concerns among the respondents (see also [9,31]).

Worry about health effects was not clearly related to proximity to the industrial site in Stenungsund. This is consistent with findings reported by Luginaah *et al*. [23]. In 2006, the lowest and highest prevalence of worry were found in the northern area of Stenungsund and in Odsmal, respectively. This could be partly explained by the large difference between the two areas in the prevalence of having children at home (17% and 42%, respectively for the northern area and for Odsmal), a factor that increased the odds of being worried about the possible health effects of industrial air pollution. The finding that women were more likely than men to be worried about the health effects of industrial air pollution may be due to concern about children’s health, since women spend more time at home when children are young. It is also possible that the high prevalence of worry in Odsmal is due to its location in the prevailing wind direction from the industries.

Annoyance due to dust and soot in the air was strongly related with worries. Such visual cues may signal that the air is polluted with potentially harmful contaminants, which may increase the concerns about health effects. This was also seen in the Canadian study [43]. Another factor which has been shown to affect health risk perceptions is trust in the industries [42,43]. We found a much lower prevalence of worry among those who were employed in the petrochemical industries compared to other gainfully employed. It is likely that those who work in these industries have a higher knowledge of the products and the industrial processes than other people, and thus a higher trust to the petrochemical companies.

### 4.4. Worry about Industrial Accidents

About 40% in the exposed area were worried about accidents associated with industrial activity. This is about three times more than in the control area. Four percent reported that they were worried daily. These numbers have not changed much between the three survey occasions. Those who were worried about the possible health effects of industrial air pollution were also substantially (OR 77) more likely to be worried about accidents due to industrial activity. This result is consistent with results reported by Luginaah *et al*. [22] and expected since an accident in the petrochemical industry or in associated transportation is likely to involve the release of hazardous chemicals into the air that could potentially affect the inhabitants’ health. We found also that worries of train accidents increased the odds of being worried about the possible health effects of industrial accidents, which is logical since dangerous goods is transported by rail through the community. 

Female sex and being gainfully employed outside the petrochemical industries were furthermore associated with worry about industrial accidents, while worry was less prevalent in those employed in petrochemical industry, who may hold a more positive attitude towards the industry. The significant influence of shorter time in the present residence on worry about industrial accidents may indicate less experience of the circumstances of living in the vicinity of petrochemical industrial plants. 

## 5. Conclusions

This longitudinal study, with three questionnaire surveys over a 15 year period (1992 to 2006) shows that living in close proximity to petrochemical industries implies a higher long-term risk of being annoyed by odour or industrial noise; about 25% annoyed in both cases. Odour annoyance decreased slightly over time, which may be due to reduced emissions in the mid-1990s. Industrial noise annoyance increased from 1992 to 2006, possibly because of more frequent episodes of burning off surplus gases. Worry about possible health effects from industrial air pollution or industrial accidents remained relatively constant over time, and worry was not clearly associated with distance to the petrochemical industry. Important risk indicators for odour annoyance and worry were female sex and having children at home. Industrial odour and noise annoyance were also related to annoyance by road traffic exhaust or road traffic noise. Worry about health effects from industrial air pollution was related to annoyance by dust and soot in the air, which indicates that visual cues may increase concern about health effects. Those employed in the petrochemical industry were less worried about health effects and accidents, probably because a more positive attitude towards this industry. 

We conclude that efforts to reduce emissions from chemical industries of air and noise pollution must be followed by communications and information from industry and authorities to strengthen confidence and reduce the prevalence of worry.
